# Determinants of digital health literacy among health professionals: Evidence from public and private hospitals in Eastern Ethiopia

**DOI:** 10.1371/journal.pone.0350299

**Published:** 2026-06-01

**Authors:** Eyerusalem Asfaw, Tilahun Shiferaw, Moti Tolera, Adisu Birhanu Weldesenbet, Mentesenot Seid

**Affiliations:** 1 Child Health and Mortality Prevention Surveillance, Hararge Health Research, Haramaya University, Haramaya, Ethiopia; 2 Department of Information Sciences, College of Computing and Informatics, Haramaya University, Haramaya, Ethiopia; 3 Capacity Building and Mentorship Program, Haramaya University, Ethiopia; 4 School of Public Health, College of Health and Medical Science, Haramaya University, Harar, Ethiopia; 5 Department of Epidemiology and Biostatistics, School of Public Health, College of Health and Medical Sciences, Haramaya University, Harar, Ethiopia; Shiraz University of Medical Sciences, IRAN, ISLAMIC REPUBLIC OF

## Abstract

**Background:**

Digital health literacy has become essential for effective clinical practice as healthcare systems increasingly adopt digital technologies. However, many low- and middle-income settings continue to face substantial gaps in digital readiness among the health workforce. Despite growing national initiatives to expand digital health in Ethiopia, evidence on digital health literacy among healthcare professionals in the eastern part remains limited. Therefore, this study aimed to assess the level of digital health literacy and its determinants among healthcare professionals in Eastern Ethiopia.

**Methods:**

A cross-sectional study was conducted from May 1–30, 2025, among 401 randomly selected healthcare professionals working in three public and private hospitals. Data were collected using a structured questionnaire based on the European Digital Competence framework and analyzed using STATA (V17.0). Multivariable logistic regression was used to identify independent predictors of digital health literacy with significance set at p < 0.05.

**Results:**

Out of 401 participants, 50.6% (95% CI: 45.62–55.62) of health professionals had adequate digital health literacy. Internet access (AOR = 3.89, 95% CI: 1.21–12.47), digital technology training (AOR = 6.24, 95% CI: 3.46–11.26), higher perceived usefulness (AOR = 2.87, 95% CI: 1.51–5.46), perceived ease of use (AOR = 1.90, 95% CI: 1.02–3.57), and good computer literacy was significant predictors of adequate digital health literacy (AOR = 3.11, 95% CI: 1.71–5.68).

**Conclusion:**

Digital health literacy among healthcare professionals in the Harari region is relatively low compared to global standards. Strengthening digital infrastructures, expanding structured digital trainings, and creating user-friendly digital environment are essential to improve digital health literacy.

## Background

Digital health literacy (DHL), an essential part of digital literacy, is the skill to find, assess, and apply digital health information to support patient care and clinical decision-making [1,2]. Over the past few years, health care systems around the world have steadily adopted digital tools like telemedicine, electronic health records (EHRs), mobile health apps, and wearable technology to improve patient care and service delivery [3,4]. For health professionals, strong digital health literacy underpins effective patient care, teamwork across disciplines, and the use of evidence-based practices [5,6].

Healthcare’s digital shift has picked up pace thanks to the rapid rise of eHealth tools, which allow real-time data access, remote doctor visits, and diagnostics supported by artificial intelligence (AI) [7,8]. However, making the most of these advances relies on healthcare workers having solid digital competencies, because when digital health literacy falls short, it can cause misinterpretation of clinical data, inefficiencies in work flow, and poorer outcomes for patients [9,10]. Despite the increasing importance of digital health literacy, not all healthcare workers are on equal footing, research shows that younger staff and those educated in tech-savvy settings tend to be more proficient than older colleagues or those with less digital exposure [11]. The absence of standardized digital health literacy training in many medical and health science curricula further continues to widen this competency gap [12,13].

In developing countries, including Ethiopia, the level of digital health literacy is still at an early stage due to limited infrastructure, inadequate access to digital tools, and insufficient training opportunities [14,15]. As health care delivery increasingly relies on digital solutions, these limitations hinder both service quality and professional efficiency, for instance during the COVID-19 pandemic the surge in Tele-health use highlighted the critical role of digital literacy; and healthcare professionals with limited digital skills faced challenges in providing remote care, resulting in compromised service quality [16–18].

Globally, studies continue to show wide variation in digital health literacy among healthcare professionals, with levels ranging from relatively high to low. For instance, 64.6% of health professionals in Catalonia and only 38.1% in Germany demonstrated adequate digital health literacy [19,20], while in Myanmar, merely 20.3% of health workers reported high information and communication technology (ICT) literacy [21]. Consistent with global trends, evidence from Ethiopia also reported digital health literacy level ranging from 43.6% to 50.1%, indicating a moderate yet suboptimal level of digital health literacy among healthcare professionals [11,12,17].

Despite these findings, evidence on digital health literacy among healthcare professionals in Harari region, Eastern Ethiopia, remains limited, leaving a knowledge gap regarding healthcare professional’s digital competencies. Generating such context-specific evidence is essential to inform targeted regional interventions, strengthen workforce digital health capacity, and support the effective integration of digital tools into routine healthcare service delivery in alignment with national digital transformation efforts. Therefore, this study aims to assess the level of digital health literacy and its determinants among healthcare professionals working in public and private hospitals in the Harari region, Eastern Ethiopia.

## Methods

### Study design, setting, and period

An institutional-based cross-sectional study was conducted from May 1–30, 2025, among healthcare professionals working in public and private hospitals in the Harari region, Eastern Ethiopia. The region is located about 526 km east of Addis Ababa and had an estimated population of 283,000 in 2023 [22].

Hospital-level healthcare services in the region are delivered through two public hospitals, one private hospital, and one federal police hospital. This study was conducted in the two public hospitals: Hiwot Fana Comprehensive Specialized University Hospital (HFCSUH) and Jugal General Hospital, and one private hospital: Harar General Hospital. The federal police hospital was excluded because it operates outside the public healthcare system and primarily serves a restricted population, limiting its comparability with facilities providing services to the general population. HFCSUH serves as a major referral and teaching hospital for Eastern Ethiopia, while Jugal and Harar general hospitals primarily provide general medical and surgical services to the local population.

### Population, sample size, and sampling procedure

The source population included all healthcare professionals working in the selected public and private hospitals. Healthcare professionals who were available during the data collection period and had been employed for at least six months were included in the study, whereas those who were on leave during data collection period were excluded.

The sample size for the first objective (to assess the level of digital health literacy) was calculated using the single population proportion formula:


$$\begin{array}{c} n=\;\frac{{\left(Z_{\alpha /\text{2}}\right)}^{\text{2}}\;x\;p(\text{1}-p)}{d^{\text{2}}} \end{array}$$

Assuming a 95% confidence level (Z_α/2_ = 1.96), 5% margin of error (d = 0.05), and an estimated prevalence of digital health literacy of 51.8% from a previous study [12], the calculated sample size was 384.

For the second objective (to identify factors associated with digital health literacy), the sample size was calculated using Epi Info version 7.2.4.0 based on the following assumptions: 95% confidence interval, 80% power, and a 1:1 ratio of exposed to un exposed groups. The proportion among exposed and unexposed groups and adjusted odd ratios were taken from a previous study [12]; including educational level (42.4% unexposed, 61.0% exposed, AOR = 4.37), access to digital technology (34.5% unexposed, 49.8% exposed, AOR = 1.89) and attitude toward digital technologies (42.3% unexposed, 54.5% exposed, AOR = 1.64). The corresponding sample sizes were 165, 232, and 361, respectively. Accordingly, the largest calculated sample size (384) from the two objectives was taken, and 10% was added to account for potential non-response, resulting in a final sample size of 422.

**Fig 1:**
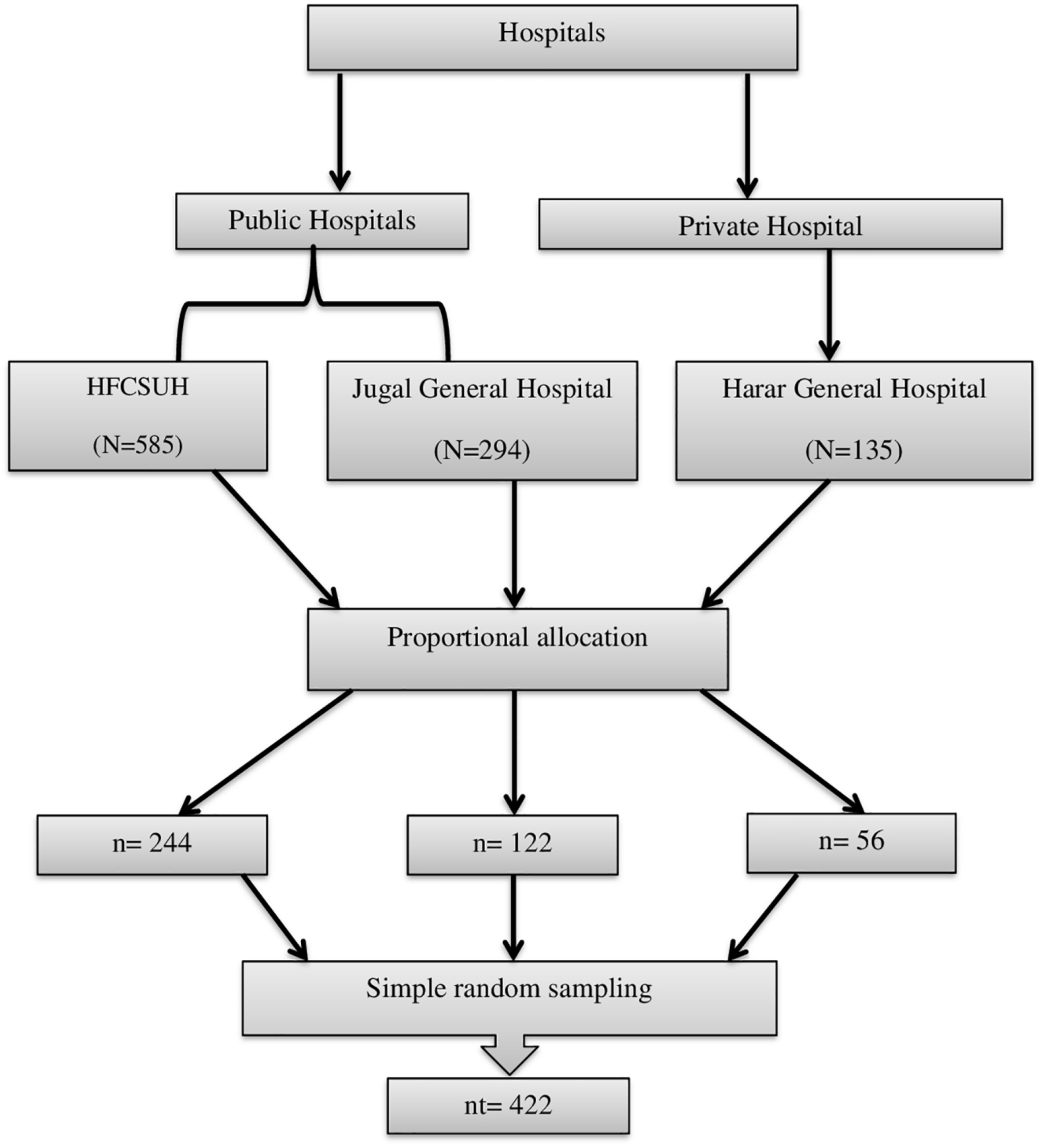
Schematic presentation of the sampling procedure for healthcare professionals in selected hospitals of Harari region, Eastern Ethiopia, 2025. HFCSUH is Hiwot Fana Comprehensive Specialized University Hospital, N = is the total number of healthcare professionals at each respective Hospital n = is the number of healthcare professionals from which data was collected in each hospital nt = is a total sample size.

All the public hospitals: HFCSUH and Jugal General Hospital, and the private hospital: Harar General Hospital, providing hospital-level healthcare services in the region were included in the study. The total sample was proportionally allocated to each hospital based on the number of healthcare professionals, and participants were then selected using simple random sampling from payroll to ensure representativeness (Fig 1).

### Variables

The dependent variable was digital health literacy level, whereas socio-demographic characteristics (sex, age professional category, educational level, work experience, economic status), technological and organizational factors (access to digital technology, internet access, training on digital technology, and computer literacy), and behavioral factors (attitudes, staff motivation, perceived usefulness, perceived ease of use) were considered as potential determinants.

### Data collection tools and procedures

Data were collected using a structured, self-administered questionnaire developed from a review of relevant literature [11,17,23,24]. The tool incorporated the European Commission’s digital competence framework, consisting of 21 items across five dimensions: information processing, content creation, communication, safety, and problem-solving [25]. Additional sections of the questionnaire, capturing socio-demographic, behavioral, and organizational factors, were adapted from validated instruments [12,26]. The questionnaire was distributed to participants during the study period, and data collection was conducted from May 1–30, 2025. Three trained BSc health science graduates collected the data at each facility, and one supervisor was assigned to closely supervise the overall data collection.

### Data quality assurance

To ensure data quality, a 2-day training was provided for both data collectors and supervisor on the study objectives, the contents of the questionnaire, and all the data collection procedures and ethical considerations to be followed throughout the data collection. Supervisor and principal investigator reviewed completed questionnaires daily to check for completeness and consistency. In addition, a pretest involving 5% of the sample size was conducted one week prior to data collection at Haramaya General Hospital, to assess the clarity, consistency of the questionnaire as well as the average time required to complete it. Necessary adjustments were made to improve the wording and clarity to the tool based on the pretest findings and experiences. Moreover, the internal reliability of the digital health literacy scale was assessed using Cronbach’s alpha (α = 0.736), indicating acceptable reliability.

### Data processing and analysis

Data were entered into EpiData version 4.6 and exported to Stata version 17 for cleaning and statistical analysis. Descriptive statistics, including frequencies, percentages, means, and standard deviations were computed to summarize participant characteristics.

Bivariable logistic regression was conducted to identify candidate variables for multivariable analysis (p < 0.25), to avoid excluding potentially important predictors. Variables meeting this criterion were entered into a multivariable logistic regression model to identify independent predictors of digital health literacy. Multicollinearity was assessed through variance inflation factor (VIF) and model fitness was assessed using the Hosmer-Lemeshow goodness-of-fit test (p = 0.53). Finally p-value <0.05 was considered statistically significant, and adjusted odd ratio (AOR) with 95% confidence interval (CI) were reported to indicate the strength and direction of associations.

### Definitions

Digital health literacy was assessed using 21 items rated on a five-point scale (Very Good to Very Poor), with the median score serving as the cutoff point; participants scoring above the median were classified as having adequate digital literacy [2,11,12]. Attitude toward digital health technologies was measured on a five-point Likert scale (Strongly Agree to Strongly Disagree), and respondents scoring at or above the median considered to have a favorable attitude [27]. Perceived usefulness and perceived ease of use were evaluated using six items each, adapted from the Technology Acceptance Model (TAM), and rated on a seven = point scale; scores above the median indicated higher perceived usefulness and ease of use [23,26]. Computer literacy, defined as the ability to effectively use computers and related technologies, was assessed using five items on a five-point Likert scale, with scores above the median classified as good computer literacy [28].

### Ethical consideration

Ethical approval was obtained from the Institutional Health Research Ethics Review Committee (IHRERC) of Haramaya University, College of Health and Medical Science (Reference No. IHRERC/198/2024). Official support letters were submitted to the regional health bureau and respective hospitals. Written informed consent was obtained from each participant after explaining the study’s purpose, ensuring voluntary participation and the right to withdraw at any time without repercussion. Confidentiality and anonymity were maintained throughout as no personal identifiers were collected.

## Results

### Socio-demographic characteristics

Of the 422 healthcare professionals approached, 401 completed and returned the questionnaire, yielding a response rate of 95.0%. The mean age of the participants was 29.37 ± 5.54 years. The majority were nurses (36.9%, n = 148), followed by medical doctors (18.2%, n = 73). Regarding educational status, the majority held a Bachelor’s degree (67.8%, n = 272), while more than half had less than five years of work experience (55.6%, n = 223) (Table 1).

**Table 1:** Socio-demographic characteristics of healthcare professionals in public and private hospitals of the Harari region, Eastern Ethiopia, 2025 (n = 401).

Socio-demographic variables	Category	Frequency	Percentage
Sex	Male	196	48.88
	Female	205	51.12
Age	29.37 ± 5.54		
Profession	Medical Doctor	73	18.20
	Health Officer	30	7.48
	Nurse	148	36.91
	Midwife	58	14.46
	Laboratory	33	8.23
	Pharmacist	31	7.73
	Other professions	28	6.98
Educational level	Diploma	82	20.45
	BSc degree	272	67.83
	Master’s degree	47	11.72
Marital status	Single	192	47.88
	Married	200	49.88
	separated or divorced	9	2.24
Religion	Orthodox	177	44.14
	Muslim	126	31.42
	Protestant	76	18.95
	Catholic	19	4.74
	Other religions	3	0.75
Work experience	<5 years	223	55.61
	5-10 years	129	32.17
	>10 years	49	12.22
Monthly Income (USD)	<5000 (< 32.51)	87	21.70
	5000-10000 (32.51- 65.01)	256	63.84
	10000-15000 (65.01 −97.78)	40	9.98
	>15000 (>97.78)	18	4.49

USD- United States dollar, other health profession includes- Health informatics, Specialists, Residents, Physiotherapist, other religion includes- Adventist and Jova

### Technological, organizational, and behavioral characteristics

Access to digital tools was reported by the majority of participants (79.1%, n = 317), and 78.8% (n = 316) had regular internet access. Approximately half (51.6%, n = 207) had received digital technology related training, and a comparable proportion (53.9%, n = 216) reported having good computer literacy. Likewise, 51.9% (n = 208) perceived digital tools as useful in their professional duties, while 53.4% (n = 214) found them user- friendly (Table 2).

**Table 2:** Technological, organizational, and behavioral characteristics of healthcare professionals in public and private hospitals of the Harari region, Eastern Ethiopia, 2025 (n = 401).

Variables	Categories	Frequency	Percentage
Access to digital technology	Yes	317	79.05
	No	84	20.95
Which digital technology do you have access to*	Desktop computer	78	37.86
	Laptop computer	128	51.65
	Smartphone	275	133.26
	Digital medical device	52	21.31
Have accessible digital technology in the workplace	Yes	223	55.61
	No	178	44.39
Internet access	Yes	316	78.80
	No	85	21.20
Frequency of internet use	Several times a day	129	40.82
	Everyday	133	42.09
	Several times a week	42	13.29
	Once a week	12	3.80
Where do you get internet access from	Private Wi-Fi and mobile data	200	63.29
	Internet cafe	9	2.85
	Workplace	107	33.86
Received training on digital technology	Yes	207	51.62
	No	194	48.38
Computer literacy	Good	216	53.87
	Poor	185	46.13
Perceived usefulness	Useful	208	51.87
	Not useful	193	48.13
Perceived ease to use	Easy	214	53.37
	Not easy	187	46.63
Motivation to use digital technology for patient care	Yes	211	52.62
	No	190	47.38

*- Multiple answer question

### Attitude towards digital health technology

Over half of the participants, 211 (52.6%), with (95% CI: 47.60–57.59) demonstrated a favorable attitude toward the use of digital health technologies in patient care. About 247 (61.6%) respondents believed that booking an appointment using digital technologies would be more convenient. Around 265 (66.1%) agreed that technology has improved healthcare. A total of 231 (57.6%) felt confident that they understand how to use technology. Additionally, 212 (52.9%) agreed that health technology reduce human error, and similarly, 241 (60.1%) expressed a positive view towards the increased use of technology in healthcare (Table 3).

**Table 3:** Attitudes of healthcare professionals toward digital health technologies in public and private hospitals of the Harari region, Eastern Ethiopia, 2025.

Attitude variables	SD n (%)	D n (%)	N n (%)	A n (%)	SA n (%)
Making an appointment on a computer or smartphone would be more convenient for me	11 (2.74)	69 (17.21)	74 (18.45)	171 (42.64)	76 (18.95)
I think using technology has improved healthcare	26 (6.48)	62 (15.46)	48 (11.97)	146 (36.41)	119 (29.68)
I really understand how to use health technology	32 (7.98)	67 (16.71)	71 (17.71)	162 (40.39)	69 (17.21)
Video and telephone appointments with my patients are as good as meeting them in person	31 (7.73)	98 (24.44)	112 (27.93)	111 (27.68)	49 (12.22)
Health technologies are easy to use	26 (6.48)	71 (17.71)	89 (22.19)	156 (38.9)	59 (14.71)
Patients and hospitals rely too much on technology	38 (9.48)	101 (25.19)	101 (25.19)	111 (27.68)	50 (12.47)
Technology could never replace real health professionals	60 (14.96)	123 (30.67)	93 (23.19)	86 (21.45)	39 (9.73)
I would like to see more use of technology in healthcare	26 (6.48	64 (15.96)	61 (15.21)	168 (41.9)	82 (20.45)
Health technology is less likely to break down, and my work will not be affected	38 (9.48)	101 (25.19)	92 (22.94)	126 (31.42)	44 (10.97)
Health technology reduces human error	30 (7.48)	66 (16.46)	93 (23.19)	143 (35.66)	69 (17.21)
The thought of using an online appointment system makes me relaxed	35 (8.73)	66 (16.46)	92 (22.94)	154 (38.4)	54 (13.47)
The thought of new developments in health technology is exciting	30 (7.48)	59 (14.71)	71 (17.71)	179 (44.64)	62 (15.46)
I often use health technology	35 (8.73)	87 (21.7)	89 (22.19)	154 (38.4)	36 (8.98)
Health technology is good for everyone	23 (5.74)	70 (17.46)	80 (19.95)	156 (38.9)	72 (17.96)
I’m confident that technology will keep the medical records private	29 (7.23)	70 (17.46)	94 (23.44)	134 (33.42)	74 (18.45)
I enjoy using health technology	27 (6.73)	64 (15.96)	95 (23.69)	149 (37.16)	66 (16.46)

SD- Strongly disagree, D- Disagree, N- Neutral, A- Agree, SA- Strongly agree

### Digital health literacy level

**Fig 2:**
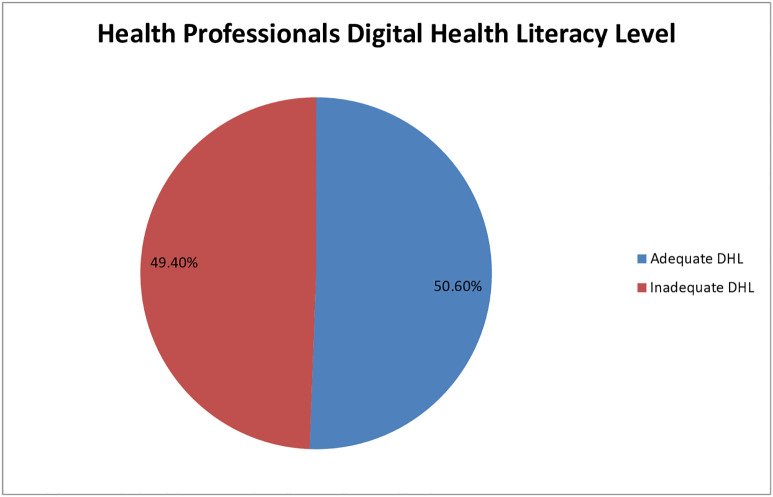
Health professionals digital health literacy level. DHL- Digital Health Literacy.

A total of 203 (50.6%) healthcare professionals (95% CI: 45.62–55.62) have adequate digital health literacy (Fig 2). Analysis of the digital health literacy subdomains showed that a total of 242 (60.4%) had adequate information processing skill. Similarly 246 (61.4%) exhibited adequate level of safety literacy. Regarding, communication, content creation, and problem solving, more than half of healthcare professionals, 212 (52.9%), 226 (56.4%), and 216 (53.9%), possessed adequate digital health literacy, respectively (Table 4).

**Table 4:** Components of digital health literacy among healthcare professionals in public and private hospitals of the Harari region, Eastern Ethiopia, 2025.

Digital health Components	Median (±SD)	Adequate n (%)	Inadequate n (%)
Information processing	9 ± 3.44	242 (60.35%)	159 (39.65%)
content creation	18 ± 5.95	226 (56.36%)	175 (43.64%)
Communication	12 ± 3.87	212 (52.87%)	189 (47.13%)
Safety	12 ± 4.0	246 (61.35%)	155 (38.65%)
Problem-solving	12 ± 3.86	216 (53.87%)	185 (46.13%)

### Factors associated with digital health literacy

In the bivariable logistic regression analysis, eleven variables including sex, educational status, monthly income, personal access to digital technology, access to digital technology at workplace, internet access, training on digital technology, attitude toward digital health technology, perceived usefulness, perceived ease of use, and computer literacy met the inclusion criterion (p < 0.25) and were entered into the multivariable logistic regression model. In the final multivariable analysis, internet access, training on digital technology, perceived usefulness, perceived ease of use, and computer literacy remained statistically significant (p < 0.05).

Health professionals with internet access were 3.89 times more likely to have adequate digital health literacy compared to those without internet access (AOR = 3.89, 95% CI: 1.21–12.47). Similarly, participants who had received training on digital technologies were 6.24 times more likely to have adequate digital health literacy compared to those without such training (AOR = 6.24, 95% CI: 3.46–11.26).

Furthermore, respondents who perceived digital technologies as useful were 2.87 times more likely to have adequate digital literacy (AOR = 2.87, 95% CI: 1.51–5.46), and those who perceived them as easy to use were 1.90 times more likely to demonstrate adequate digital literacy (AOR = 1.90, 95% CI: 1.02–3.57). Additionally, health care professionals with a good computer literacy were 3.11 times more likely to have adequate digital health literacy than those with poor computer literacy (AOR = 3.11, 95% CI: 1.71–5.68) (Table 5).

**Table 5:** Multivariable logistic regression analysis of factors associated with digital health literacy among healthcare professionals in public and private hospitals of the Harari region, Eastern Ethiopia, 2025.

Variables		Digital literacy level		COR (95% CI)	AOR (95% CI)	P-value
		Adequate	Inadequate			
Sex	Female	95 (23.69)	110 (27.43)	1	1	
	Male	108 (26.93)	88(21.95)	1.42 (0.96-2.11)	1.14 (0.65-1.99	0.647
Educational level	Diploma	16 (3.99)	66 (16.46)	1	1	
	BSc degree	149 (37.16)	123 (30.67)	4.99 (2.75-9.07)	2.35 (0.90-6.12)	0.081
	Master’s degree	38 (9.48)	9 (2.24)	17.42 (7.02-43.20)	2.55 (0.69-9.45)	0.161
Monthly Income (USD)	<5000 (< 32.51)	26 (6.48)	61 (15.21)	1	1	
	5000-10000 (32.51- 65.01)	131 (32.67)	125 (31.17)	2.46 (1.46- 4.14)	0.68 (0.28-1.64)	0.392
	10000-15000 (65.01 −97.78)	30 (7.48)	10 (2.5)	7.04 (3.00-16.47)	1.40 (0.38-5.14)	0.609
	>15000 (>97.78)	16 (3.99)	2 (0.5)	18.77 (4.02-87.55)	0.98 (0.15-6.50)	0.979
Access to digital technology	No	11 (2.7)	73 (18.2)	1	1	
	Yes	192 (47.9)	125 (31.2)	10.19 (5.20-19.97)	1.13 (0.37-3.45)	0.828
Access to digital technology in workplace	No	105 (26.2)	73 (18.2)	1	1	
	Yes	98 (24.4)	125 (31.2)	0.55 (0.37- 0.81)	0.94 (0.53- 1.67)	0.841
Internet access	No	10 (2.49)	75 (18.70)	1	1	
	Yes	193 (48.13)	123 (30.67)	11.77 (5.86- 23.64)	3.89 (1.21-12.47)	0.022*
Training on digital technology	No	51 (12.7)	143 (35.7)	1	1	
	Yes	152 (37.9)	55 (13.7)	7.75 (4.97- 12.08)	6.24 (3.46-11.26)	0.000***
Attitude	Unfavorable	64 (16.0)	126 (31.4)	1	1	
	favorable	139 (34.7)	72 (18.0)	3.8 (2.51- 5.75)	0.96 (0.53-1.73)	0.892
Perceived usefulness	Not useful	49 (12.22)	144 (35.91)	1	1	
	Useful	154 (38.4)	54 (13.47)	8.38 (5.35- 13.12)	2.87 (1.51-5.46)	0.001**
Perceived ease of use	Not easy	49 (12.2)	138 (34.4)	1	1	
	Easy	154 (38.4)	60 (15.0)	7.23 (4.65-11.24)	1.90 (1.02-3.57)	0.045*
Computer literacy	Poor	51 (12.72)	134 (33.42)	1	1	
	Good	152 (37.91)	64 (15.96)	6.24 (4.04-9.64)	3.11 (1.71-5.68)	0.000***

USD- United States dollar, 1- reference category, superscript *** indicates p < 0.001, ** indicates p < 0.01, * indicates p < 0.05 and statistically significant categories at multivariable logistic analysis at 5% level of significant, CI-confidence interval, COR- crude odd ratio, AOR- adjusted odd ratio

## Discussion

This study investigated digital health literacy (DHL) among healthcare professionals in public and private hospitals in the Harari region and explored the key factors associated with its variation. The observed proportion of adequate DHL 50.6% (95% CI: 45.62–55.62) is comparable to several Ethiopian studies 51.8% in Gonder [12], 49.7% in Northwest Amhara [11], 50.4% in the Amhara region [23], and 53.8% in Addis Ababa [24].

However, the DHL level observed in this study appears relatively lower when compared with findings from high income settings such as Vietnam (76.3%) [29], Turkey (70%) [30], Australia (70–80%) [7], and Jordan (84%) [31]. This discrepancy could be explained by the presence of setting based contextual differences like stronger digital infrastructure, more reliable internet connectivity, and greater integration of digital health tools within healthcare systems [32]. Furthermore, health workers in high-income settings typically receive more structured digital training and have higher exposure to health information systems, which actively promote their good digital competency [7,33]. Importantly, this indicates that improving DHL in low- and middle-income settings requires more than individual-level interventions as it necessitates coordinated health system strengthening, including infrastructure development, governance, and sustained technical support.

Conversely, the DHL level reported in this study was slightly higher when compared to other studies from Ethiopia (43.6%) [17] and (45.8%) [34]. A possible explanation for this difference may lie in the study population where particularly inclusion of hospital-based healthcare professionals with frequent exposure to digital system [23,35]. This reveals health system inequity with uneven distribution of digital health gains and this limits effectiveness of digital health intervention in underserved areas. Internet access was found to be significant determinant of DHL, health professionals with regular connectivity had significantly higher odds of adequate DHL compared to those without. This finding aligns with prior studies where internet availability was linked with improved digital competencies among healthcare workers [1,12,23,2]. A plausible explanation could be that reliable connectivity expands opportunities for both formal and informal learning such as accessing guidelines, webinars, and continuing professional development, which makes connectivity to be translated consistently into measurable gains in DHL [36,37]. This implies investment in connectivity is of paramount benefits in improving access to information, skill development and practical utility.

In addition, this study found that those who had undergone training were considerably more likely to demonstrate adequate DHL. This finding is consistent with evidence from previous studies in northern Ethiopia [12,23]. A potential reason for this might be attributed to its direct benefit in bridging knowledge gaps, enhancing confidence in using digital tools and reducing resistance to adopting new technologies [32,38]. Moreover study have highlighted that structured digital training innervations significantly improve health workers’ digital health literacy, and readiness to adopt different digital health tools and decision- support systems [15,35]. However, the persistence of moderate DHL levels despite training exposure indicates that current training approaches may lack depth, continuity, or practical orientation. This implies the need for more context-specific, competency-based, and continuous training models rather than one-time interventions.

Likewise, higher perceived usefulness of digital health technologies was significantly associated with higher DHL. This finding was corroborated by previous studies conducted in Ethiopia, southeastern Poland, and various European countries [23,39,40]. Similarly, greater perceived ease of use of digital health technologies was also linked to higher DHL, in line with reports from studies in Ethiopia [17,23]. These associations might be resonate with the theoretical underpinnings of the Technology Acceptance Model (TAM) and have been empirically found in other contexts [23]. These findings could also possibly explained by the fact that health professionals positive perceived usefulness and find them user-friendly can foster openness, motivation, and proactive engagement with digital tools making them to strength their digital health literacy and further explore, adopt, and integrate digital tools into routine patient care [40–42]. This highlights improving DHL is not solely the responsibility of users; it also depends on designing systems that are intuitive, relevant, and aligned with clinical workflows. Poorly designed systems may inadvertently act as barriers, discouraging engagement and limiting skill development [35,43].

Computer literacy also showed strong association with higher odd of achieving adequate DHL. Similar observation was demonstrated by different studies conducted in different part of Ethiopia [17,23]. The possible rational behind this association might be explained by the fact that computer literacy provides the necessary baseline for digital health competencies such as interpreting digital health data, and engaging with e-health systems [44,45]. Moreover, evidences from WHO and other studies indicates that strengthening computer literacy among health workers is a pragmatic and cost-effective entry point for building DHL [15,38,46]. This study demonstrated that parallel investments in infrastructure, capacity building, and system integration are crucial to achieve their intended impact towards DHL.

### Strengths and limitations

A key strength of this study lies in its multicenter design, encompassing three major hospitals, both public and private, that serve as a referral center for the region and neighboring populations. This enhances the representativeness and generalizability of the findings. However, as a cross-sectional study, it cannot establish causal relationships between the identified factors and digital health literacy. Additionally, reliance on self-reported data may introduce social desirability bias as participants could have overestimated their responses.

## Conclusion

This study showed that about half of healthcare professionals have adequate digital health literacy, highlighting ongoing gaps in the readiness of health professionals to effectively use and implement digital tools in regional clinical practice. A relatively low level of digital health literacy compared to global standards underscore the need for targeted interventions to support ongoing digital health transformations. Having internet access, prior training in digital technology, perceived usefulness and ease of use of digital tools, and strong computer literacy emerged as significant predictors of higher digital health literacy. Addressing these factors requires a coordinated, multi-level effort that integrates capacity building with improvements in digital infrastructure, reliable connectivity, and the usability of digital tools.

### Recommendation

In the current digital era, improving the digital literacy of healthcare professionals is essential for successfully implementing digital health technologies. Policy-level investment and coordinated efforts by hospital administrations, regional health bureaus, and national authorities should focus on strengthening digital infrastructure, enhancing computer literacy, expanding structured training programs, and ensuring that digital tools are perceived as useful and easy to use. Future research should also include lower-level healthcare facilities and adopt mixed-method approaches to gain more comprehensive insights.

## Supporting information

S1 File Asfaw_2025_DHL_V01.Excel Dataset. (XLS)

S2 File Asfaw 2025 DHL Questionnaire. (DOCX)

## Abbreviations

AOR: Adjusted Odds Ratio
CI: Confidence Interval
COVID-19: Corona virus Disease
COR: Crude odds ratio
DHL: Digital Health Literacy
e-HEALS: Electronic Health Literacy Scale
EMRs: Electronic Medical Records
FMOH: Federal Ministry of Health
HCWs: Health Care Workers
HPs: Health Professionals
ICT: Information Communication Technology
IT: Information Technology
SD: Standard Deviation.

## References

[1] Arias López MDP, Ong BA, Borrat Frigola X, Fernández AL, Hicklent RS, Obeles AJT, et al. Digital literacy as a new determinant of health: a scoping review. PLOS Digit Health. 2023;2(10):e0000279. doi: 10.1371/journal.pdig.0000279 37824584 PMC10569540

[2] Zhao B-Y, Huang L, Cheng X, Chen T-T, Li S-J, Wang X-J, et al. Digital health literacy and associated factors among internet users from China: a cross-sectional study. BMC Public Health. 2024;24(1):908. doi: 10.1186/s12889-024-18324-0 38539176 PMC10976739

[3] Holt KA, Karnoe A, Overgaard D, Nielsen SE, Kayser L, Røder ME, et al. Differences in the Level of Electronic Health Literacy Between Users and Nonusers of Digital Health Services: an exploratory survey of a group of medical outpatients. Interact J Med Res. 2019;8(2):e8423. doi: 10.2196/ijmr.8423 30950809 PMC6473204

[4] Saner H. Digital health implementation: how to overcome the barriers?. London, England: SAGE Publications Sage UK; 2019. 10.1177/2047487319848222 31046444

[5] Islam SMS, Maddison R. Digital health approaches for cardiovascular diseases prevention and management: lessons from preliminary studies. Mhealth. 2021;7:41. doi: 10.21037/mHealth-2020-6 34345618 PMC8326947

[6] Norman CD, Skinner HA. eHealth literacy: essential skills for consumer health in a networked world. J Med Internet Res. 2006;8(2):e9. doi: 10.2196/jmir.8.2.e9 16867972 PMC1550701

[7] Kuek A, Hakkennes S. Healthcare staff digital literacy levels and their attitudes towards information systems. Health Informatics J. 2020;26(1):592–612. doi: 10.1177/1460458219839613 30983476

[8] Longhini J, Rossettini G, Palese A. Digital health competencies among health care professionals: systematic review. J Med Internet Res. 2022;24(8):e36414. doi: 10.2196/36414 35980735 PMC9437781

[9] Ngusie HS, Kassie SY, Chereka AA, Enyew EB. Healthcare providers’ readiness for electronic health record adoption: a cross-sectional study during pre-implementation phase. BMC Health Serv Res. 2022;22(1):282. doi: 10.1186/s12913-022-07688-x 35232436 PMC8889777

[10] Brørs G, Larsen MH, Hølvold LB, Wahl AK. eHealth literacy among hospital health care providers: a systematic review. BMC Health Serv Res. 2023;23(1):1144. doi: 10.1186/s12913-023-10103-8 37875882 PMC10599073

[11] Shiferaw KB, Tilahun BC, Endehabtu BF, Gullslett MK, Mengiste SA. E-health literacy and associated factors among chronic patients in a low-income country: a cross-sectional survey. BMC Medical Inform Decision Mak. 2020;20(1):181. 10.1186/s12911-020-01202-1 PMC7407428 32762745

[12] Tegegne MD, Tilahun B, Mamuye A, Kerie H, Nurhussien F, Zemen E, et al. Digital literacy level and associated factors among health professionals in a referral and teaching hospital: an implication for future digital health systems implementation. Front Public Health. 2023;11:1130894. doi: 10.3389/fpubh.2023.1130894 37113180 PMC10126829

[13] van der Vaart R, Drossaert C. Development of the digital health literacy instrument: measuring a broad spectrum of health 1.0 and health 2.0 skills. J Med Internet Res. 2017;19(1):e27. doi: 10.2196/jmir.6709 28119275 PMC5358017

[14] Chetty K, Qigui L, Gcora N, Josie J, Wenwei L, Fang C. Bridging the digital divide: measuring digital literacy. Economics. 2018;12(1). doi: 10.5018/economics-ejournal.ja.2018-23

[15] Organization WH. Digital health literacy key to overcoming barriers for health workers, WHO study says. 2023. https://www.who.int/europe

[16] Platis C, Mollas N, Psomiadi ME, Theodorou P. Investigation of digital health information literacy and the relationship between burnout and job satisfaction of health professionals in three public hospitals during Covid-19 pandemic. Int J Lat Res Human Soc Sci. 2022;5:89–99.

[17] Ahmed MH, Guadie HA, Ngusie HS, Teferi GH, Gullslett MK, Hailegebreal S, et al. Digital Health literacy during the COVID-19 pandemic among health care providers in resource-limited settings: cross-sectional study. JMIR Nurs. 2022;5(1):e39866. doi: 10.2196/39866 36301671 PMC9665171

[18] Turnbull SL, Dack C, Lei J, Aksu I, Grant S, Lasseter G, et al. Barriers and facilitators to use of digital health tools by healthcare practitioners and their patients, before and during the COVID-19 pandemic: a multimethods study. BMJ Open. 2024;14(3):e080055. doi: 10.1136/bmjopen-2023-080055 38448080 PMC10916085

[19] Reixach E, Andrés E, Sallent Ribes J, Gea-Sánchez M, Àvila López A, Cruañas B, et al. Measuring the digital skills of catalan health care professionals as a key step toward a strategic training plan: digital competence test validation study. J Med Internet Res. 2022;24(11):e38347. doi: 10.2196/38347 36449330 PMC9752462

[20] Pfob A, Sidey-Gibbons C, Schuessler M, Lu S-C, Xu C, Dubsky P, et al. Contrast of digital and health literacy between IT and health care specialists highlights the importance of multidisciplinary teams for digital health-A pilot study. JCO Clin Cancer Inform. 2021;5:734–45. doi: 10.1200/CCI.21.00032 34236897

[21] Oo HM, Htun YM, Win TT, Han ZM, Zaw T, Tun KM. Information and communication technology literacy, knowledge and readiness for electronic medical record system adoption among health professionals in a tertiary hospital, Myanmar: a cross-sectional study. PLoS One. 2021;16(7):e0253691. doi: 10.1371/journal.pone.0253691 34197506 PMC8248629

[22] Agency ES. Population size by sex, region, zone and wereda. Ababa, Ethiopia: Ethiopian Statistical Services; 2023.

[23] Chereka AA, Demsash AW, Ngusie HS, Kassie SY. Digital health literacy to share COVID-19 related information and associated factors among healthcare providers worked at COVID-19 treatment centers in Amhara region, Ethiopia: a cross-sectional survey. Inform Med Unlocked. 2022;30:100934. doi: 10.1016/j.imu.2022.100934 35441087 PMC9010014

[24] Assaye BT, Kassa M, Belachew M, Birhanu S, Worku A. Association of digital health literacy and information-seeking behaviors among physicians during COVID-19 in Ethiopia: a cross-sectional study. Digit Health. 2023;9:20552076231180436. doi: 10.1177/20552076231180436 37312956 PMC10259119

[25] Carretero GS, Vuorikari R, Punie Y. DigComp 2.1: the digital competence framework for citizens with eight proficiency levels and examples of use. 2017.

[26] Godoe P, Johansen TS. Understanding adoption of new technologies: technology readiness and technology acceptance as an integrated concept. J Eur Psychol Students. 2012;3:38. doi: 10.5334/jeps.aq

[27] Currie M, Philip LJ, Roberts A. Attitudes towards the use and acceptance of eHealth technologies: a case study of older adults living with chronic pain and implications for rural healthcare. BMC Health Serv Res. 2015;15:162. doi: 10.1186/s12913-015-0825-0 25888988 PMC4415301

[28] Andualem M, Kebede G, Kumie A. Information needs and seeking behaviour among health professionals working at public hospital and health centres in Bahir Dar, Ethiopia. BMC Health Serv Res. 2013;13:534. doi: 10.1186/1472-6963-13-534 24373296 PMC3877973

[29] Do BN, Tran TV, Phan DT, Nguyen HC, Nguyen TTP, Nguyen HC, et al. Health literacy, ehealth literacy, adherence to infection prevention and control procedures, lifestyle changes, and suspected covid-19 symptoms among health care workers during lockdown: online survey. J Med Internet Res. 2020;22(11):e22894. doi: 10.2196/22894 33122164 PMC7674138

[30] Şahïn D, Firat S, Gezïcï N. The effect of healthcare professionals’ digital literacy and knowledge of telemedicine on perception of telemedicine. Inter J Health Manag Tourism. 2023. doi: 10.31201/ijhmt.1326835

[31] Shudayfat T, Bani Hani S, Al Qadire M. Assessing digital health literacy level among nurses in Jordanian hospitals. Electron J Gen Med. 2023;20(5):em525. doi: 10.29333/ejgm/13466

[32] Organization WH. Advancing the responsible use of digital technologies in global health: A report of the WHO Science Council: World Health Organization; 2025.

[33] Yew SQ, Trivedi D, Adanan NIH, Chew BH. Facilitators and barriers to the implementation of digital health technologies in hospital settings in lower- and middle-income countries since the onset of the covid-19 pandemic: scoping review. J Med Internet Res. 2025;27:e63482. doi: 10.2196/63482 40053793 PMC11926458

[34] Daka DW, Muluemebet AW, Abdi KL, Nigatu W, Animut N, Abraham L. Health extension workers’ digital literacy and their attitude towards community-level electronic health information systems in Tiro Afata Woreda, Southwest Ethiopia. 2022.

[35] Kasaye MD, Kebede N, Kalayou MH, Kebede SD, Molla A. Digital health literacy and associated factors among health professionals during the outbreak of corona virus pandemic in Ethiopia: a systematic review and meta-analysis. Digit Health. 2024;10:20552076241271799. doi: 10.1177/20552076241271799 39148812 PMC11325474

[36] Borges do Nascimento IJ, Abdulazeem H, Vasanthan LT, Martinez EZ, Zucoloto ML, Østengaard L, et al. Barriers and facilitators to utilizing digital health technologies by healthcare professionals. NPJ Digit Med. 2023;6(1):161. doi: 10.1038/s41746-023-00899-4 37723240 PMC10507089

[37] Rossner SS, Gizaw M, Getachew S, Getachew E, Destaw A, Negash S, et al. Health care professionals’ knowledge, attitude, practice, and infrastructure accessibility for e-learning in ethiopia: cross-sectional study. JMIR Med Educ. 2025;11:e65598. doi: 10.2196/65598 40997320 PMC12463343

[38] Alotaibi N, Wilson CB, Traynor M. Enhancing digital readiness and capability in healthcare: a systematic review of interventions, barriers, and facilitators. BMC Health Serv Res. 2025;25(1):500. doi: 10.1186/s12913-025-12663-3 40186200 PMC11969766

[39] Borges do Nascimento IJ, Abdulazeem H, Vasanthan LT, Martinez EZ, Zucoloto ML, Østengaard L, et al. Barriers and facilitators to utilizing digital health technologies by healthcare professionals. NPJ Digit Med. 2023;6(1):161. doi: 10.1038/s41746-023-00899-4 37723240 PMC10507089

[40] Burzyńska J, Rękas M, Januszewicz P. Evaluating the psychometric properties of the ehealth literacy scale (eHEALS) among polish social media users. Int J Environ Res Public Health. 2022;19(7):4067. doi: 10.3390/ijerph19074067 35409753 PMC8997910

[41] Davis FD. Perceived usefulness, perceived ease of use, and user acceptance of information technology. MIS Quarterly. 1989;13(3):319–40. doi: 10.2307/249008

[42] Hussain A, Zhiqiang M, Li M, Jameel A, Kanwel S, Ahmad S, et al. The mediating effects of perceived usefulness and perceived ease of use on nurses’ intentions to adopt advanced technology. BMC Nurs. 2025;24(1):33. doi: 10.1186/s12912-024-02648-8 39789568 PMC11716174

[43] Tubaishat A. Perceived usefulness and perceived ease of use of electronic health records among nurses: application of technology acceptance model. Inform Health Soc Care. 2018;43(4):379–89. doi: 10.1080/17538157.2017.1363761 28920708

[44] GDHP. Global digital health partnership; digital health literacy toolkit. 2025.

[45] Foundational digital literacy curriculum for community health workers. PATH; 2021.

[46] Organization WH. Global strategy on digital health 2020-2025. 2021.

