# Supplementary material for: Determinants of digital health literacy among health professionals: Evidence from public and private hospitals in Eastern Ethiopia
Source: PLoS One. 2026 Jun 1;21(6):e0350299. doi: 10.1371/journal.pone.0350299 (PMC13225363; doi:10.1371/journal.pone.0350299)
Supplement: S2 File — (DOCX) [file pone.0350299.s002.docx]

# ANNEXES

# Participant Information Sheet and informed voluntary consent form in English for the Hospital Director

**Introduction:** My name is Eyerusalem Asfaw I am studying for my Master’s degree at Haramaya University, College of Health and Medical Sciences. I kindly request you to give me your attention to explain you about the study.

**1. The study /project title:** Assessing digital health literacy and its determinants among health professionals in public and private hospitals in Harar region, eastern Ethiopia.

**2. Aim of the study:** The study finding will help to plan training programs to improve digital health literacy of health care providers thereby to increase the performance of health care organizations. Moreover, this study aims to write a thesis as a partial requirement for the fulfillment of a Master’s Program in health service management.

**3. Procedure and duration:** Questionnaire will be provided which in returned with pertinent data that is helpful for the study. There are questions to answer in which the health care providers will fill the questionnaire by themselves and will take about 20 minutes.

**4. Risk and benefits:** The risk of participating in this study is very minimal, only taking some of their time. There would not be any direct payment for participating in this study. But the findings from this research may reveal important information for the local and national health planners.

**5. Confidentiality:** The information that will be collected from participants will be confidential. There will be no information that will identify participant in particular. The findings of the study will be general for health care providers and will not reflect anything particular to an individual. The questionnaire will be coded to exclude showing names. No reference will be made in oral or written reports that could link participants to the research.

**6. Rights:** Participating in this study is fully voluntary. The participants have the right to declare to participate or not in this study. If they decide to not participate, they have the right to withdraw from the study at any time and this will not label them for any loss of benefits which they otherwise are entitled. They do not have to answer any question that they do not want to answer and the institution has the right to stop this study from being conducted in the institution if any misdeeds and unethical procedures are observed.

**7. Contact address:** If there are any questions or inquires any time about the study or the procedures, please contact: Eyerusalem Asfaw eyerusasfew7@gmail.com or Mobile -; 0946038010 as well as contact address of the responsible Institutional Health Research Ethics Review Committee (IHRERC) at office phone 0254662011 or P.O.Box 235, Harar.

**8. Declaration of informed voluntary consent:** I have read the participant information sheet. I have clearly understood the purpose of the research, the procedure, the risks and benefits, issues of confidentiality, the rights of participating and contact address for any queries. I have been given the opportunity to ask questions for things that may have been unclear. I was informed that participants have the right to withdraw from the study at any time or not to answer any question that they do not want. I am also informed that the hospital has the right to stop this study from being conducted in their institution if any misdeeds and unethical procedures are observed during the data collection process in the hospital premises.

Therefore, I declare my voluntary consent on behalf of the hospital for this study to be conducted with my initials (signature).

Name and signature of hospital director: _________________ Date______________

Name and signature of investigator: ______________________Date______________

N.B

This is signed face to face in the presence of the data collector.

Please provide a copy of this signed consent to the participant.

##

## Participant Information Sheet and informed voluntary consent form in English for the Participant (for participant: ages >18 years).

**1. Introduction**

My name is M/r/s------------------------------------.I am working as a data collector for the study being conducted in this public and private hospitals by Eyerusalem Asfaw, who is studying for her Master’s degree at Haramaya University, College of Health and Medical Sciences. I kindly request you to give me your attention to explain you about the study and being selected as the study participant.

1. **The study /project title:** Assessing digital health literacy and its determinants among health professionals in public and private hospitals in Harar region, eastern Ethiopia.

**3. Aim of the study:** The study finding will help to plan training programs to improve digital health literacy of health care providers thereby to increase the performance of health care organizations. Moreover, this study aims to write a thesis as a partial requirement for the fulfillment of a Master’s Program in health service management.

**4. Procedure and duration:** I will give you a questionnaire to provide me information with pertinent data that is helpful for the study. There are questions to answer where you will fill the questionnaire by yourself and it will take about 20 minutes. So I kindly request you to spare me this time.

**5. Risk and benefits:** The risk of participating in this study is very minimal, only taking some of their time. There would not be any direct payment for participating in this study. But the findings from this research may reveal important information for the local and national health planners.

**6. Confidentiality:** The information you will provide us will be confidential. There will be no information that will identify you in particular. The findings of the study will be general for the study community and will not reflect anything in particular of individual person. The questionnaire will be coded to exclude showing names. No reference will be made in oral or written reports that could link participants to the research.

**7. Rights:** Participating in this study is fully voluntary. You have the right to declare to participate or not in this study. If you decide to participate, you have the right to withdraw from the study at any time and this will not label you for any loss of benefits which you otherwise are entitled. You do not have to answer any question that you do not want to answer.

**8. Contact address:** If there are any questions or inquires any time about the study or the procedures, please contact: Eyerusalem Asfaw eyerusasfew7@gmail.com or Mobile -**;** 0946038010 and Health Research Ethics Review Committee (IHRERC) at office phone 0254662011 or P.O.Box 235, Harar.

**9. Declaration of informed voluntary consent:** I have read/ was read to me the participant information sheet. I have clearly understood the purpose of the research, the procedures, the risks and benefits, issues of confidentiality, the rights of participating and the contact address for any queries. I have been given the opportunity to ask questions about things that may have been unclear. I was informed that I have the right to withdraw from the study at any time or not to answer any question that I do not want. Therefore, I declare my voluntary consent to participate in this study with my initials (signature).

Name and signature of participant: __________________________Date ____________

Name and Signature of Data Collector__________________________ Date_________

N.B

This is signed face to face in the presence of the data collector.

Please provide a copy of this signed consent to the participant

## 7.3. Self-Administered Questionnaire in English

Name of hospital ___________

**Part I: Socio- demographic characteristics**

**Instruction:** Please circle the number in the option you choose & fill in the blank space that best describe you on the right side of the table.

| No | Questions | Response categories |
| --- | --- | --- |
| 1 | Sex | 1.Male  2. Female |
| 2 | Age (in Years) | ___________ |
| 3 | Profession | 1. Medical Doctor  2. Health Officer  3. Nurse  4. Midwife  5. Laboratory  6. Pharmacist  7. Other, specify___________ |
| 4 | Educational Qualification | 1. Diploma  2. BSc degree  3. MSc degree  4. Other, specify____________ |
| 5 | Marital status | 1.Single  2.Married  3.Separated or divorced |
| 6 | Religion | 1. Orthodox  2. Muslim  3. Protestant  4. Catholic  5. Other(specify)_______ |
| 7 | Work experience | 1. <5 years  2. 5-10 years  3. >10 years |
| 8 | Monthly income (Ethiopian birr) | 1. <5000  2. 5000-10,000  3. 10,000-15,000  4. >15,000 |

**Part II: Technological and organizational related questions**

| S. No | Questions | Response categories |
| --- | --- | --- |
| 9 | Do you have access to digital technology? | 1. Yes 2. No |
| 10 | Which digital technology do you have access to? | 1. Desktop computer 2. Laptop computer 3. Smartphone 4. Digital medical device |
| 11 | Is there any accessible digital technology in your workplace? | 1. Yes 2. No |
| 12 | Do you have an internet access? | 1. Yes 2. No |
| 13 | If you say yes to the question no 12, where do you get access to the internet | 1. Privet Wi-if and mobile data 2. Internet cafe 3. Workplace |
| 14 | How often do use Internet | 1. several times a day 2. Everyday 3. several times a week 4. once a week |
| 15 | Have you ever revived training on digital technology? | 1. Yes 2. No |
| 16 | Do you have motivation to use digital technology for patient care? | 1. Yes 2. No |

**Part III: Level digital health literacy**

**Instruction:** How would you rate your skills for using each of the followings? Please put a tick (√) in the box at the appropriate spot: ‘Very Good’, ‘Good, ‘Acceptable’, ‘Poor’, or ‘Very Poor’.

| No | Questions | Very poor | Poor | Acceptable | Good | Very good |
| --- | --- | --- | --- | --- | --- | --- |
| 17 | How do you describe your ability on searching and browsing data, information and digital content for patient care? |  |  |  |  |  |
| 18 | How do you describe your ability on evaluating data, information and digital content for patient care? |  |  |  |  |  |
| 19 | How do you describe your ability on managing data, information and digital content for patient care? |  |  |  |  |  |
| 20 | How do you describe your status on interacting through digital technologies? |  |  |  |  |  |
| 21 | What is your status on sharing information through digital technologies? |  |  |  |  |  |
| 22 | What is your level of engagement with patients through digital technologies? |  |  |  |  |  |
| 23 | What is your level of collaboration through digital technologies for patient care? |  |  |  |  |  |
| 24 | How do you describe your respect and appropriate way of communication on the internet? |  |  |  |  |  |
| 25 | How do you describe your ability to manage different digital characters? |  |  |  |  |  |
| 26 | How do you describe your ability on developing digital contents for patient care? |  |  |  |  |  |
| 27 | How do you describe yourself on integrating and re-explaining digital contents? |  |  |  |  |  |
| 28 | What is your status on copyright and licenses applications when using digital technologies? |  |  |  |  |  |
| 29 | How do you describe your ability on Programming using digital technologies? |  |  |  |  |  |
| 30 | How do you describe yourself on Protecting technological devices? |  |  |  |  |  |
| 31 | How do you describe yourself on Protecting personal data and privacy? |  |  |  |  |  |
| 32 | How do you describe yourself at Protecting health and well-being using digital technologies? |  |  |  |  |  |
| 33 | How do you describe yourself at Protecting the environment using digital technologies? |  |  |  |  |  |
| 34 | How do you describe your ability on Solving technical problems using digital technologies? |  |  |  |  |  |
| 35 | How do you describe yourself on identifying needs and give technological responses? |  |  |  |  |  |
| 36 | How do you describe your ability of creativity using digital technologies? |  |  |  |  |  |
| 37 | How do you describe yourself on identifying digital skill gaps? |  |  |  |  |  |

**Part IV: Attitude towards digital health technology**

**Instruction:** Please indicate the extent to which you agree or disagree with the following statements by putting a tick (√) in the box at the appropriate spot: ‘Strongly Agree’, ‘Agree’, ‘Neutral’, ‘Disagree’ or ‘Strongly Disagree’.

| S. No | Variables | Strongly Agree’ | Agree | Neutral | Disagree | Strongly Disagree’ |
| --- | --- | --- | --- | --- | --- | --- |
| 38 | Making an appointment on a computer or smartphone would be more convenient for me |  |  |  |  |  |
| 39 | I think using technology has improved healthcare |  |  |  |  |  |
| 40 | I really understand how to use health technology |  |  |  |  |  |
| 41 | Video and telephone appointments with my patients are as good as meeting them in person |  |  |  |  |  |
| 42 | Health technologies are easy to use |  |  |  |  |  |
| 43 | Patients and hospitals rely too much on technology |  |  |  |  |  |
| 44 | Technology could never replace real health professionals |  |  |  |  |  |
| 45 | I would like to see more use of technology in healthcare |  |  |  |  |  |
| 46 | Health technology is less likely to break down, and my work will not be affected |  |  |  |  |  |
| 47 | Health technology reduces human error |  |  |  |  |  |
| 48 | The thought of using an online appointment system makes me relaxed |  |  |  |  |  |
| 49 | The thought of new developments in health technology is exciting |  |  |  |  |  |
| 50 | I often use health technology |  |  |  |  |  |
| 51 | Health technology is good for everyone |  |  |  |  |  |
| 52 | I'm confident that technology will keep the medical records private |  |  |  |  |  |
| 53 | I enjoy using health technology |  |  |  |  |  |

**Part V: Perceived usefulness and Perceived ease of use**

**Instruction:** Please indicate your level of frequency of using each of the followings by putting a tick (√) in the box at the appropriate spot: ‘extremely unlikely’, ‘quite unlikely’, ‘slightly unlikely’, ‘neither’, ‘slightly likely’, ‘quite likely’ and ‘extremely likely’.

| **Perceived usefulness** | | | | | | | | | |
| --- | --- | --- | --- | --- | --- | --- | --- | --- | --- |
| S. No | Variables | | extremely unlikely | quite unlikely | slightly unlikely | neither | slightly likely’ | quite likely | extremely likely |
| 54 | digital technologies enables me to accomplish tasks more quickly | |  |  |  |  |  |  |  |
| 55 | digital technologies improves my job performance | |  |  |  |  |  |  |  |
| 56 | Using digital technologies increases my productivity | |  |  |  |  |  |  |  |
| 57 | Using digital technologies enhances my effectiveness on the job | |  |  |  |  |  |  |  |
| 58 | Using digital technologies makes it easier to do my job | |  |  |  |  |  |  |  |
| 59 | Overall, I find digital technologies useful in my job | |  |  |  |  |  |  |  |
| **Perceived ease of use** | | | | | | | | | |
| 60 | Learning to operate the digital technologies is easy for me |  | |  |  |  |  |  |  |
| 61 | I find it easy to get the digital technologies to do what I want it to do |  | |  |  |  |  |  |  |
| 62 | Usage of the digital technologies is clear and understandable |  | |  |  |  |  |  |  |
| 63 | I find it cumbersome to use the digital technologies |  | |  |  |  |  |  |  |
| 64 | It is easy for me to remember how to perform tasks using digital technologies |  | |  |  |  |  |  |  |
| 65 | Overall, I find the digital technologies easy to use |  | |  |  |  |  |  |  |

**Part VI: Computer literacy**

**Instruction:** Please indicate the extent to which you agree or disagree with the following statements by putting a tick (√) in the box at the appropriate spot: ‘Strongly Agree’, ‘Agree’, ‘Neutral’, ‘Disagree’ or ‘Strongly Disagree’.

| S. No | Questions | Strongly Agree | Agree | Neutral | Disagree | Strongly Disagree’ |
| --- | --- | --- | --- | --- | --- | --- |
| 66 | I understand basic functions of computer hardware components |  |  |  |  |  |
| 67 | I understand basic functions of computer software components |  |  |  |  |  |
| 68 | I can use keyboard shortcuts |  |  |  |  |  |
| 69 | I have a personal homepage or a personal portfolio on the web |  |  |  |  |  |
| 70 | I find it easy to learn something by reading it on the computer screen |  |  |  |  |  |
| 71 | I find it easy to learn something by watching it on the computer screen |  |  |  |  |  |

## 
